# Persistent gut barrier damage and commensal bacterial influx following eradication of *Giardia* infection in mice

**DOI:** 10.1186/1757-4749-5-26

**Published:** 2013-08-30

**Authors:** Tzu-Ling Chen, Shin Chen, Hsiu-Wei Wu, Tsung-Chun Lee, Yen-Zhen Lu, Li-Ling Wu, Yen-Hsuan Ni, Chin-Hung Sun, Wei-Hsuan Yu, Andre G Buret, Linda Chia-Hui Yu

**Affiliations:** 1 Graduate Institute of Physiology, National Taiwan University College of Medicine, Taipei, Taiwan; 2 Department of Internal Medicine, National Taiwan University College of Medicine, Taipei, Taiwan; 3 Department of Pediatrics, National Taiwan University College of Medicine, Taipei, Taiwan; 4 Graduate Institute of Parasitology, National Taiwan University College of Medicine and Hospital, Taipei, Taiwan; 5 Graduate Institute of Biochemistry and Molecular Biology, National Taiwan University College of Medicine and Hospital, Taipei, Taiwan; 6 Department of Biological Sciences, Inflammation Research Network, University of Calgary, Calgary, Alberta, Canada

**Keywords:** Giardiasis, Post-infective intestinal dysfunction, Epithelial barrier, Tight junction, Bacterial endocytosis

## Abstract

**Background:**

Recent studies of *Giardia lamblia* outbreaks have indicated that 40–80% of infected patients experience long-lasting functional gastrointestinal disorders after parasitic clearance. Our aim was to assess changes in the intestinal barrier and spatial distribution of commensal bacteria in the post-clearance phase of *Giardia* infection.

**Methods:**

Mice were orogastrically inoculated with *G. lamblia* trophozoites (strain GS/M) or pair-fed with saline and were sacrificed on post-infective (PI) days 7 (colonization phase) and 35 (post-clearance phase). Gut epithelial barrier function was assessed by Western blotting for occludin cleavage and luminal-to-serosal macromolecular permeability. Gut-associated, superficial adherent, and mucosal endocytosed bacteria were measured by agar culturing and were examined by fluorescence *in situ* hybridization. Intracellular bacteria cultured from isolated mucosal cells were characterized by 16S rDNA sequencing. Neutrophil-specific esterase staining, a myeloperoxidase activity assay, and enzyme-linked immunosorbent assays for cytokine concentrations were used to verify intestinal tissue inflammation.

**Results:**

Tight junctional damage was detected in the intestinal mucosa of *Giardia*-infected mice on PI days 7 and 35. Although intestinal bacterial overgrowth was evident only during parasite colonization (PI day 7), enhanced mucosal adherence and endocytosis of bacteria were observed on PI days 7 and 35. Multiple bacterial strains, including *Bacillus*, *Lactobacillus*, *Staphylococcus*, and *Phenylobacterium*, penetrated the gut mucosa in the post-infective phase. The mucosal influx of bacteria coincided with increases in neutrophil infiltration and myeloperoxidase activity on PI days 7 and 35. Elevated intestinal IFNγ, TNFα, and IL-1β levels also were detected on PI day 35.

**Conclusions:**

*Giardia*-infected mice showed persistent tight junctional damage and bacterial penetration, accompanied by mucosal inflammation, after parasite clearance. These novel findings suggest that the host’s unresolved immune reactions toward its own microbiota, due to an impaired epithelial barrier, may partly contribute to the development of post-infective gut disorders.

## Background

The protozoan parasite, *Giardia lamblia* (synonymous with *G. duodenalis, G. intestinalis*), is the most commonly identified agent causing waterborne diarrheal disease worldwide. *G. lamblia* is a strictly lumen-dwelling pathogen characterized by trophozoites that adhere to and colonize the small intestine, resulting in symptoms of diarrhea, abdominal pain, and weight loss in humans. In some cases, giardiasis can present as a self-limiting acute infection. Recent evidence suggests that acute *Giardia* infection may lead to the development of chronic functional gastrointestinal disorders, such as post-infectious irritable bowel syndrome (IBS) and functional dyspepsia, by unknown mechanisms [1,2]. Follow-up studies of a waterborne outbreak of giardiasis in Norway have reported that 40–80% of patients experienced abdominal symptoms consistent with IBS after the eradication of parasites [3-6]. The percentage of patients who developed post-infective gut disorders following giardiasis is higher than those following bacterial or viral gastroenteritis (3–30%) [1,7].

Intestinal pathologies in giardiasis include villous atrophy, brush border shortening, and epithelial barrier disruption in infected humans and animals [8-10]. Tight junctional damage has been demonstrated in epithelial cell cultures following exposure to live *Giardia* or trophozoite lysates [10-12]. Besides direct parasitic effects, host immune factors responding to *Giardia* infection are responsible, in part, for increased intestinal permeability [8,13-16]. It remains unclear whether these gut barrier defects are corrected after parasitic expulsion.

The gut lumen is the largest reservoir of commensal bacteria in the human body. It harbors an estimated 100 trillion bacteria that gradually increase along proximal to distal segments, reaching their highest number in the colon [17-19]. Bacterial overgrowth in the small intestine was documented in an early clinical report of giardiasis in which 8 of 17 patients showed increased bacterial counts in jejunal aspirates [20]. Whether colonic bacterial numbers are affected by parasitic infection of the small intestine remains unknown. Although *Giardia* parasites do not disseminate beyond the epithelial layer during colonization, we speculate that infection with *Giardia* may trigger long-lasting changes in the spatial distribution of commensal microorganisms and induce microbial penetration and inflammatory responses in the gut mucosa during the post-clearance phase.

## Results

### I. Time course of Giardia infection in mice

**Figure 1:**
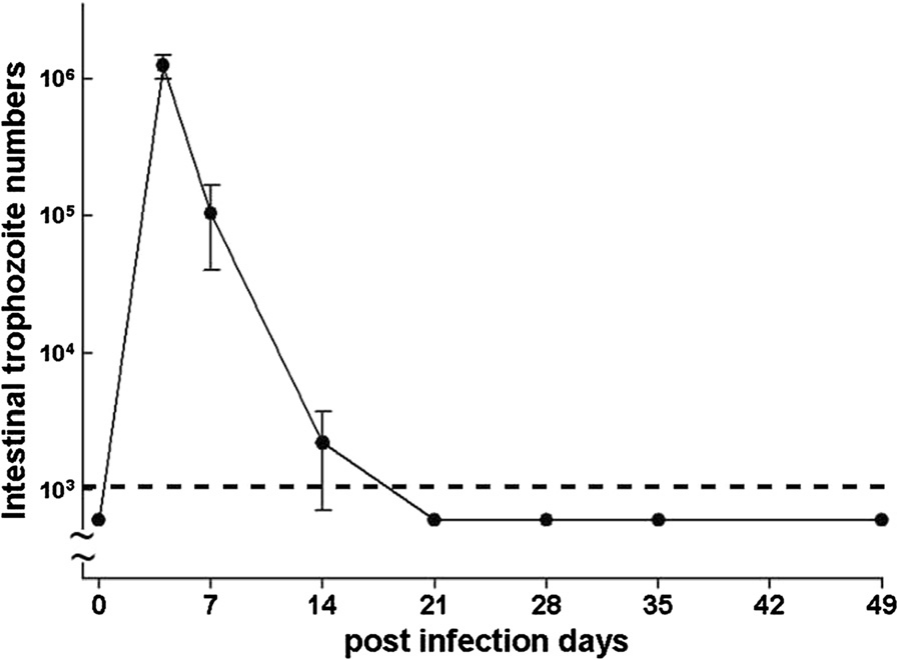
**Time course of *G. lamblia *infection in mice.** Trophozoite numbers in the mouse small intestine were determined at the indicated times. *G. lamblia* colonization peaked on PI days 4–7, and clearance was observed by days 14–21. Values represent mean ± SEM. The dashed line indicates the detection limit of trophozoite counts. Subsequent experiments were conducted on PI day 7 (colonization phase) and PI day 35 (post-clearance phase). *n* = 6–8/group. *P < 0.05 *vs.* day 0.

Trophozoite numbers in the small intestine were determined on various days after oral inoculation of mice with *G. lamblia* strain GS/M. Parasite colonization peaked on post-infective (PI) days 4–7 and was cleared by PI days 14–21 (Figure 1). No parasites were detected in control mice inoculated with saline (data not shown). Therefore, subsequent experiments were conducted with PI day 7 regarded as the “colonization phase” and PI day 35 as the “post-clearance phase”.

### II. Persistent tight junctional damage during parasite colonization and post-clearance of Giardia infection

**Figure 2:**
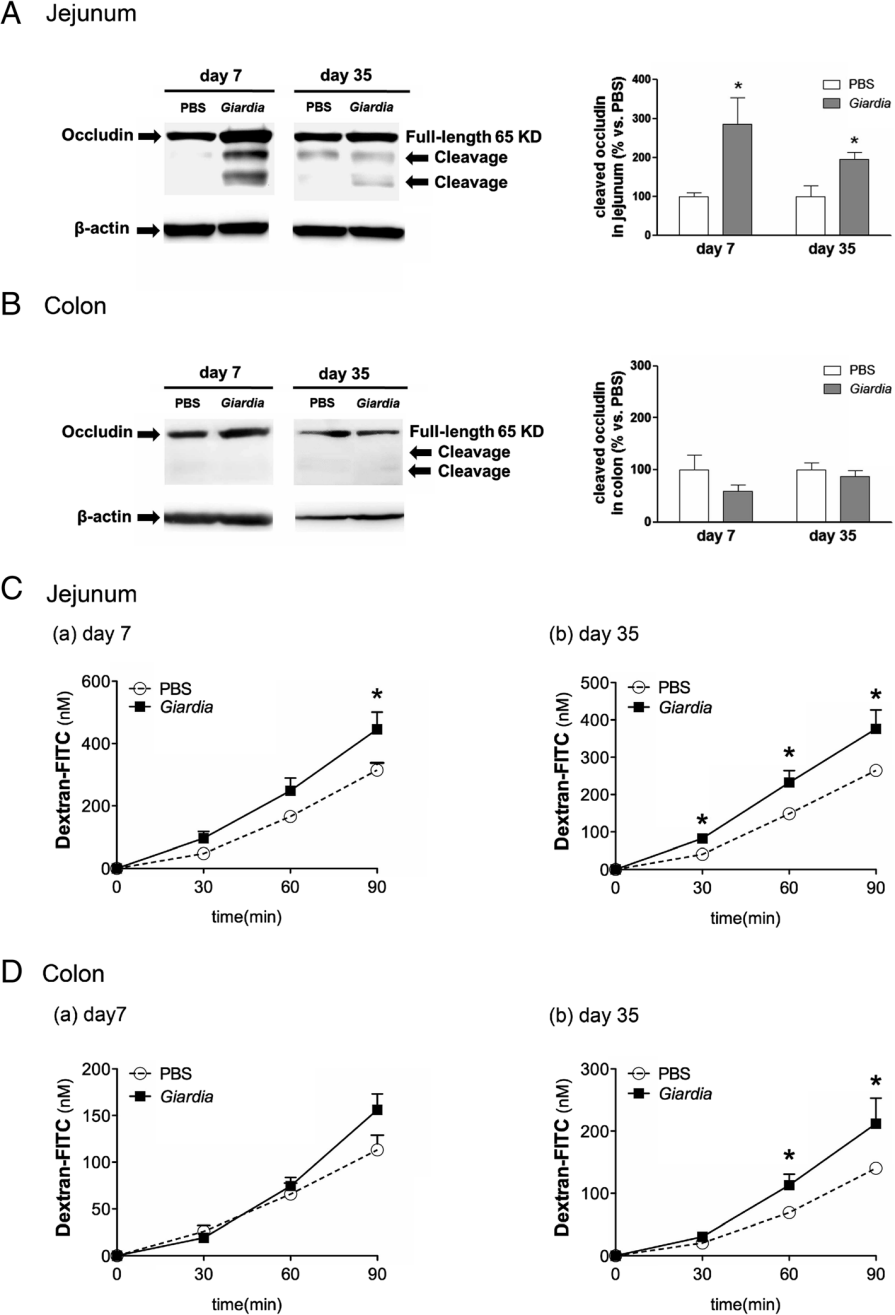
**Increased occludin cleavage in jejunum samples of *Giardia*-infected mice during parasite colonization and post-clearance.** The tight junctional integrities of intestinal tissues of *Giardia*-infected mice and PBS control groups were examined on PI days 7 and 35. **(A)** Western blots depicting occludin cleavage in the jejunal mucosa of *Giardia*-infected mice, but not saline controls, on PI days 7 and 35. Representative blots from at least two individual experiments. *n* = 4/group. *P < 0.05 *vs.* PBS. **(B)** No signs of occludin cleavage were observed in the colonic mucosa of infected mice or saline controls on PI days 7 and 35. Representative blots from at least two individual experiments. *n* = 4/group. **(C)** Luminal-to-serosal dextran flux in jejunal tissues of infected mice on PI day 7 **(a)** and day 35 **(b)**. *n* = 6/group. *P < 0.05 *vs.* PBS. **(D)** Luminal-to-serosal dextran flux in colonic tissues of infected mice on PI day 7 **(a)** and day 35 **(b)**. *n* = 6/group. *P < 0.05 *vs.* PBS.

Previous studies have described tight junctional disruption and increased epithelial permeability in *Giardia*-infected intestines [10-12]. We observed increased occludin cleavage in the jejunal mucosa on PI days 7 and 35 following *Giardia* infection (Figure 2A). No sign of occludin damage was observed in colonic samples at either time points (Figure 2B). In addition, the luminal-to-serosal dextran flux was increased in jejunal tissues of infected mice on PI days 7 and 35 (Figure 2C-a and -b). Colonic tissues did not display increased epithelial permeability on PI day 7, but a significant increase in permeability was detected during the post-infective phase on PI day 35 (Figure 2D-a and -b). Normal mucosal morphology was observed in jejunum and colon samples from infected mice on PI days 7 and 35 (Additional file 1: Figure S1).

### III. Post-infective adherence and penetration of commensal bacteria

**Figure 3:**
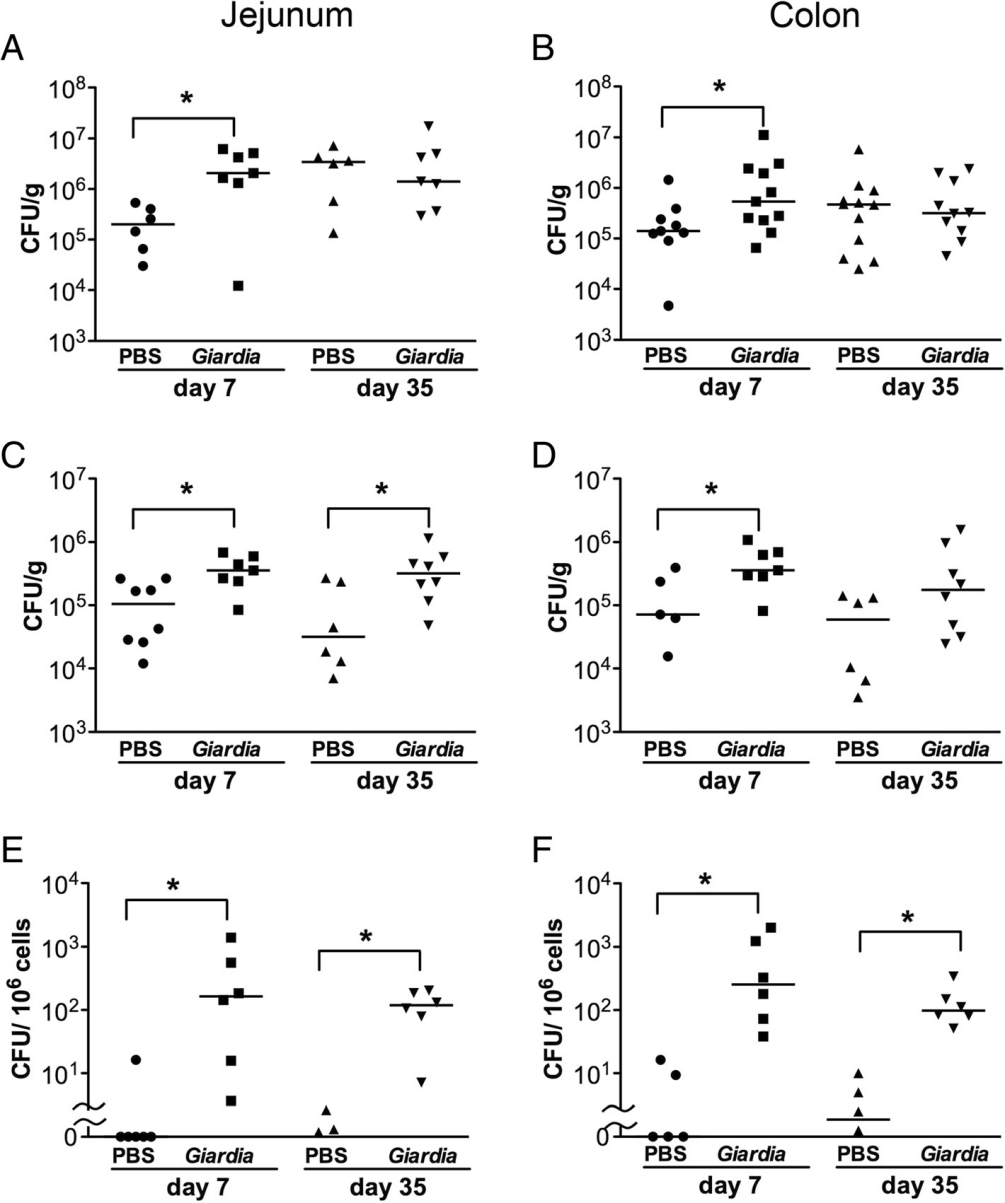
**Enhanced bacterial adherence and penetration in *Giardia*-infected mouse intestines that persist into the post-clearance phase.** Gut-associated **(A** and **B)**, superficial adherent **(C** and **D)**, and mucosal endocytosed **(E** and **F)** bacterial counts were determined on PI days 7 and 35 by aerobic culturing. Bacterial counts were examined in segments of jejunum **(A**, **C**, **E)** and colon **(B**, **D**, **F)** in each mouse. Each data point corresponds to one animal. Bars indicate the median bacterial counts. **(A** and **B)***n* =9–12/group; **(C–F)***n* = 6–8/group. *P < 0.05 *vs.* PBS.

Gut-associated bacterial counts in the jejunum and colon samples of *Giardia*-infected mice were significantly elevated compared with saline controls on PI day 7, as determined by aerobic culturing (Figures 3A and 3B). In the post-clearance phase on PI day 35, no difference in gut-associated bacterial numbers was detected between saline controls and infected mice in either jejunum or colon samples by aerobic culturing (Figures 3A and 3B). A similar trend in bacterial numbers was observed when anaerobic culture methods were used (Additional file 2: Figure S2).

The spatial distributions of superficial and intracellular bacteria were evaluated in jejunum and colon samples. A 3 to 4-times increase in superficial bacterial counts in the jejunum was observed in *Giardia*-infected mice compared with saline controls on PI days 7 and 35 (Figure 3C), suggesting persistent bacterial adherence in the small intestines. An increase in superficial bacterial counts also was identified in the colons of infected mice versus saline controls on PI day 7; this increase failed to reach statistical significance on PI day 35 (Figure 3D).

Endocytosed bacterial counts in gut mucosal cells were assessed using a gentamycin resistance assay to avoid contamination with luminal or superficial bacteria. Higher numbers of mucosal endocytosed bacteria were measured in *Giardia*-infected mice compared with saline controls on PI days 7 and 35 in jejunum and colon samples (Figures 3E and 3F). Negligible levels of bacteria were detected in liver and spleen tissues of *Giardia*-infected animals and saline controls on PI days 7 and 35 (data not shown).

**Figure 4:**
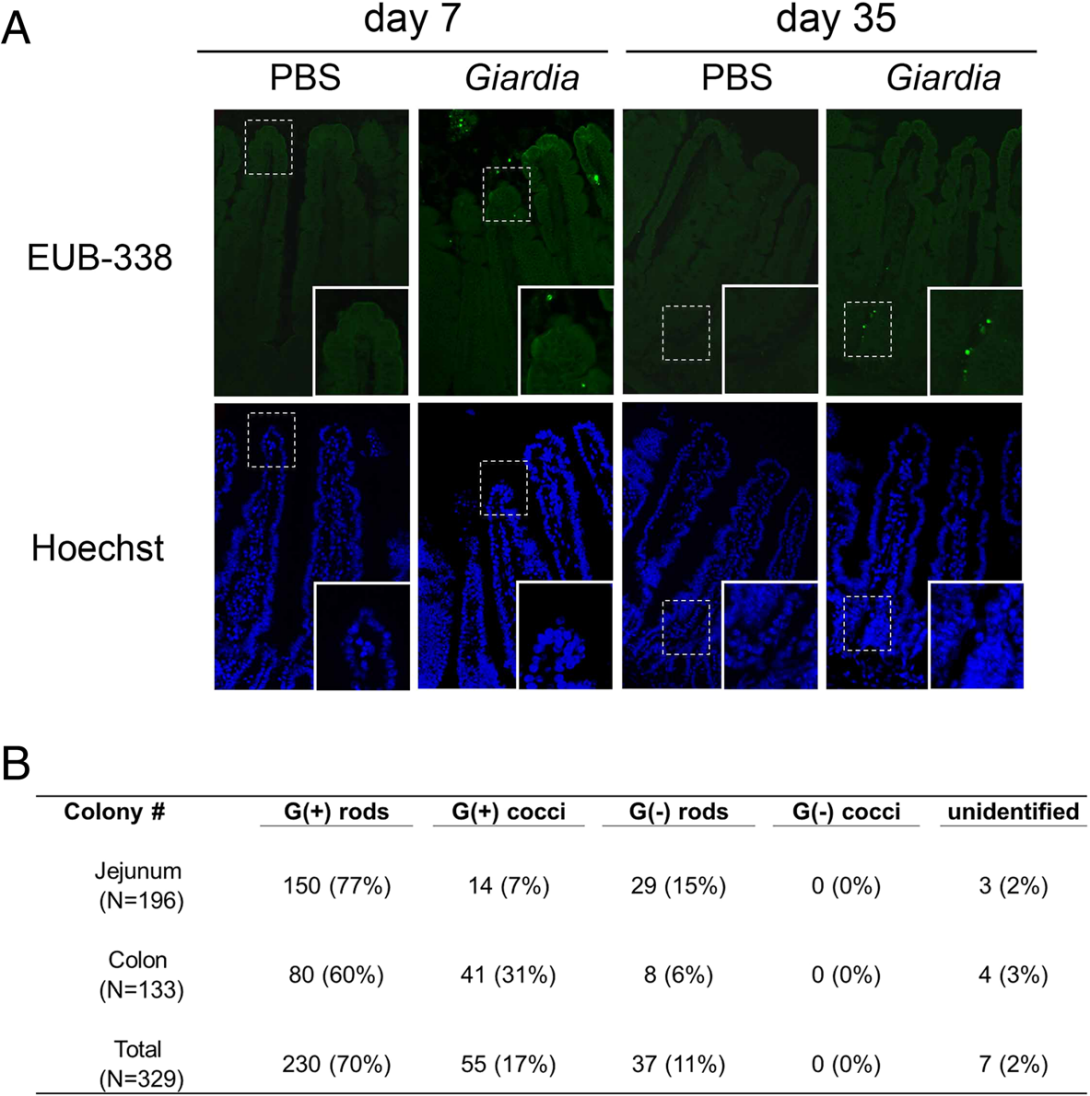
**Internalization of multiple bacterial strains into the gut mucosa during the post-infective phase. (A)** Representative photomicrographs of bacterial internalization into the post-infective gut mucosa (magnification ×200). Jejunal segments of *Giardia*-infected mice and saline controls were subjected to FISH with universal bacterial DNA probes. Bacteria (green, top panel) were detected in the epithelial layer and lamina propria of *Giardia*-infected mice but not in saline controls. A higher magnification of mucosal bacteria is shown in the insets. Hoechst-stained cell nuclei (blue, bottom panel) are shown for orientation. Photoimages were obtained from at least four mice per group. **(B)** Classification of internalized bacterial strains in the post-infective phase. Bacterial colonies cultured from lysed mucosal cells of jejunal (*N* = 196) and colonic (*N* = 133) segments were subjected to Gram staining. The percentages of colonies classified as Gram-positive or -negative rods or cocci are shown.

Using *in situ* hybridization, bacteria were visualized in the epithelial layer and lamina propria of infected mouse intestinal tissues on PI days 7 and 35. In contrast, bacteria were not detected in the tissues of saline controls at either time point (Figure 4A). To assess the strains and diversity of internalized microbes, bacterial colonies derived from lysed mucosal cells of post-infective mice were subjected to Gram staining or DNA extraction. The percentages of colonies classified as Gram-positive rods, Gram-positive cocci, Gram-negative rods, and Gram-negative cocci were 70, 17, 11, and 0%, respectively (Figure 4B). Two percent of the bacterial colonies remained unclassified and unidentified. According to 16S rDNA sequencing, multiple strains of bacteria (*i.e. Bacillus*, *Lactobacillus, Staphylococcus*, and *Phenylobacterium)* were present in mucosal cells during the post-infective phase.

### IV. Mucosal phagocytic activation during colonization and post-clearance of *Giardia* infection

**Figure 5:**
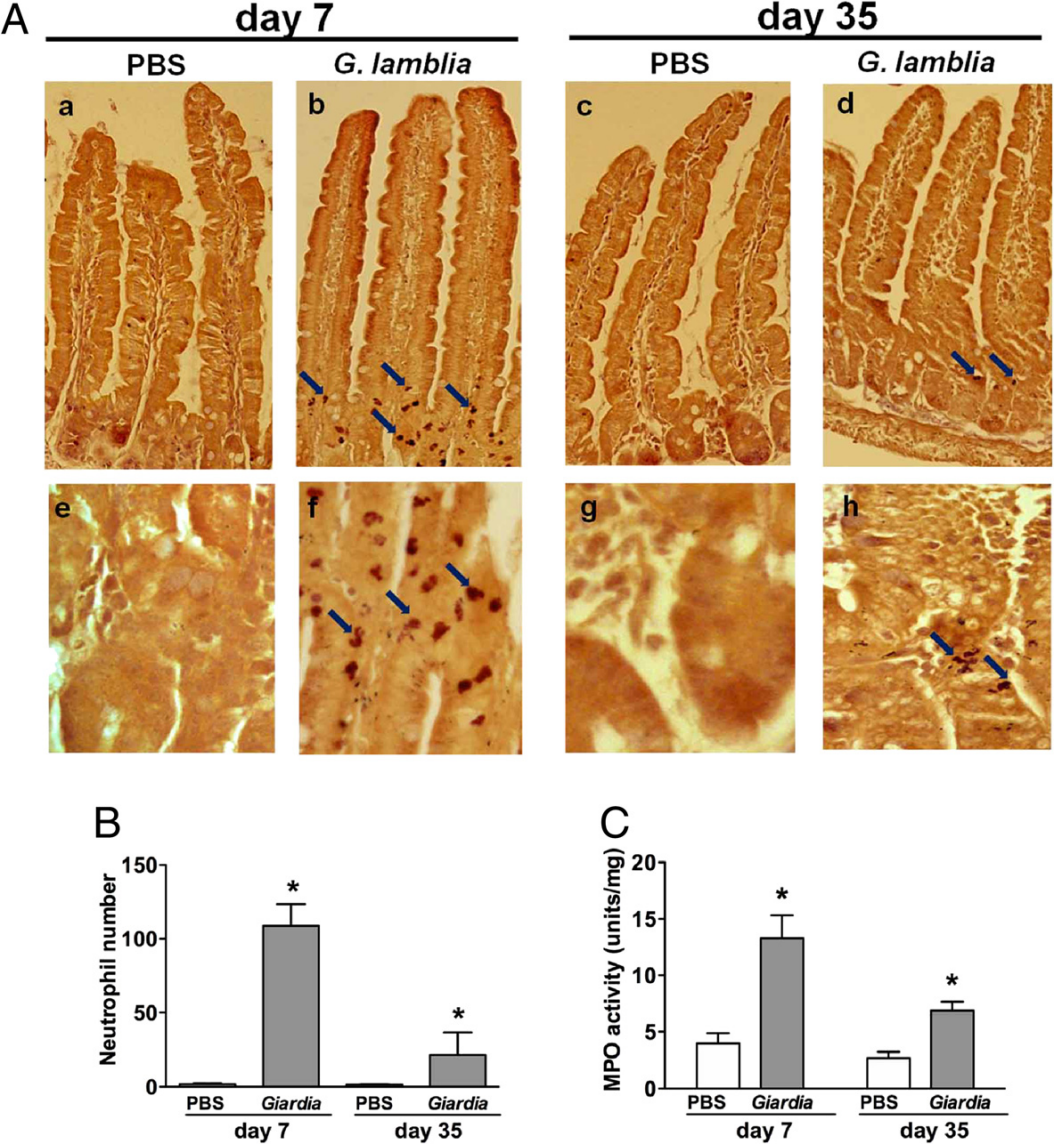
**Neutrophil infiltration into the gut mucosa during colonization and post-clearance phases in *Giardia*-infected mice. (A)** High numbers of neutrophils (arrows) were noted in the lamina propria of Giardia-infected mouse jejunum samples **(b**, **d)** compared to saline controls **(a**, **c)** on PI days 7 and 35. Enlarged images are shown in the bottom panels **(e**, **f**, **g**, **h)**. **(B)** Quantification results demonstrate increased numbers of mucosal neutrophils in infected mice compared with saline controls on PI days 7 and 35. **(C)** Increased levels of MPO activity were observed in the small intestines of infected mice compared with saline controls on PI days 7 and 35. n = 6–8/group. *P < 0.05 *vs.* PBS.

Although *Giardia* does not invade the mucosa, we speculated that abnormal bacterial influx could trigger the infiltration and activation of mucosal neutrophils. By neutrophil-specific esterase staining, high numbers of polymorphonuclear neutrophils were observed in the lamina propria of *Giardia*-infected mouse jejunum samples on PI days 7 and 35 (Figure 5A). In contrast, very few neutrophils were detected in saline control groups (Figure 5A). Mucosal neutrophil numbers were significantly elevated in *Giardia*-infected mice compared with saline controls on PI day 7 (108.8 ± 14.8 *vs.* 1.7 ± 0.5, *P* < 0.05), and numbers remained elevated during the post-clearance phase on PI day 35 (21.5 ± 15.0 *vs.* 1.6 ± 0.3, *P* < 0.05) (Figure 5B). The infiltration status of neutrophils correlated with MPO activity, in which increased levels were observed in the small intestines of infected mice on PI days 7 and 35 (Figure 5C).

**Figure 6:**
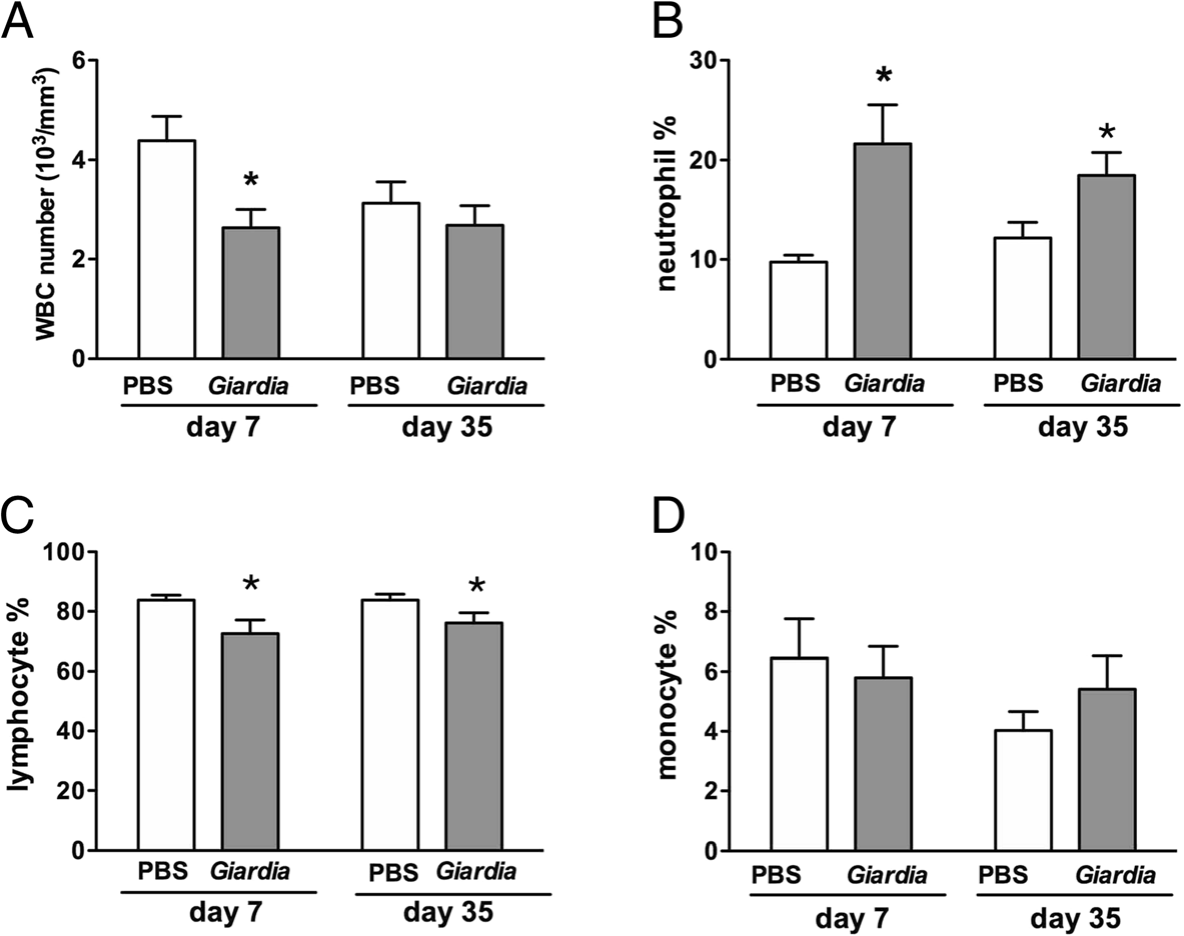
**Total and differential blood leukocyte counts in *Giardia*-infected mice.** Total blood leukocyte counts **(A)** and the percentages of neutrophils **(B)**, lymphocytes **(C)**, and monocytes **(D)** in infected mice and saline controls on PI days 7 and 35. *n* = 6–8/group. *P < 0.05 *vs.* PBS.

Total blood leukocyte counts were lower in *Giardia*-infected mice than in saline controls on PI day 7, whereas the values in the two groups were comparable on PI day 35 (Figure 6A). However, the percentage of neutrophils in the blood leukocyte populations of infected mice was two-times higher than in saline controls on PI day 7 (21.6 ± 3.9% *vs.* 9.7 ± 1.6%, *P* < 0.05) and on PI day 35 (20.3 ± 1.4% *vs.* 11.0 ± 1.1%, *P* < 0.05) (Figure 6B). The percentage of lymphocytes was lower in infected mice than in saline controls on PI days 7 and 35 (Figure 6C), and comparable percentages of monocytes were observed between infected and control mice (Figure 6D).

### V. Increased proinflammatory cytokine levels in post-infective intestinal tissues

**Figure 7:**
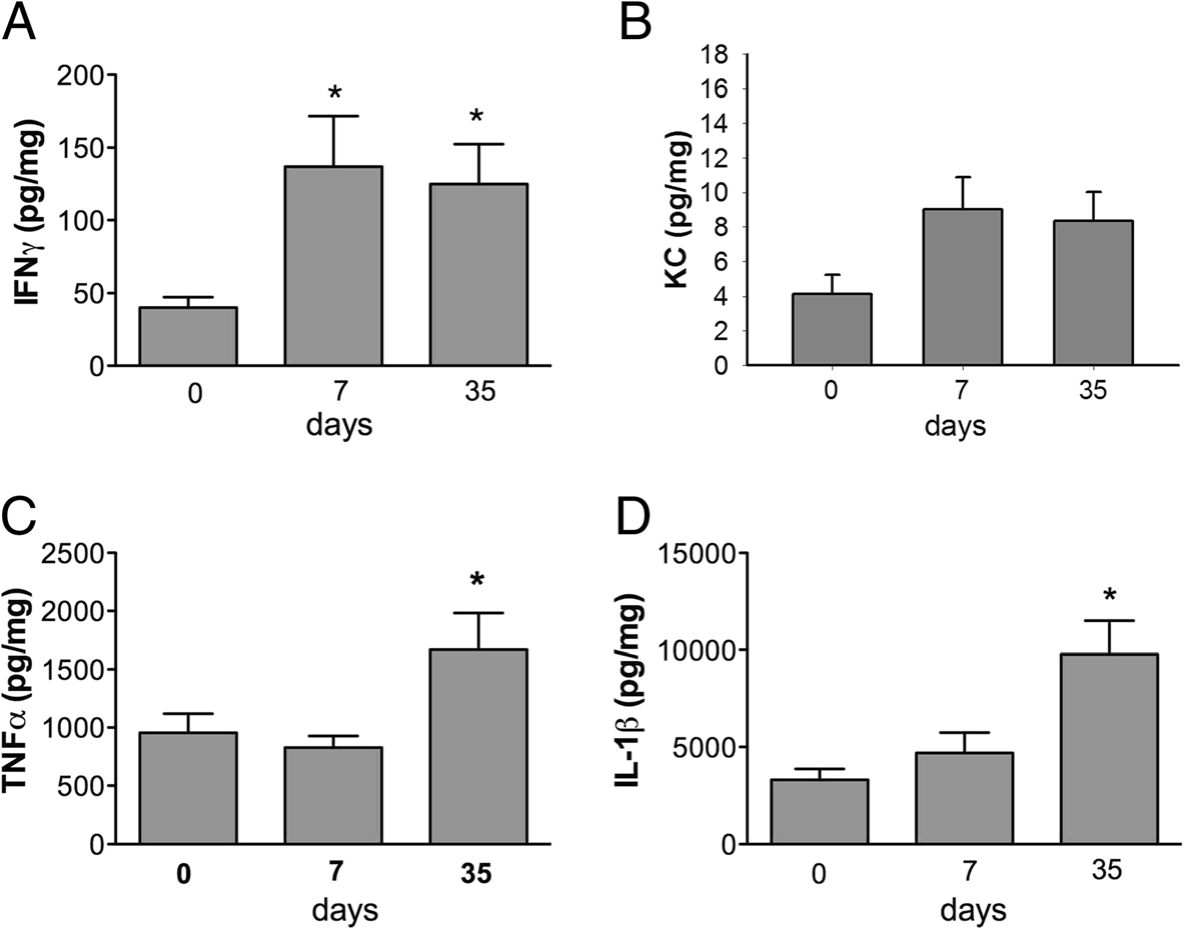
**Elevated proinflammatory cytokines in intestinal tissues after parasite clearance.** Levels of IFNγ **(A)**, KC **(B)**, TNFα **(C)**, and IL-1β **(D)** were determined in intestinal mucosal samples of uninfected mice (day 0), infected mice (day 7), and post-infective mice (day 35). *n* =5–6/group. *P < 0.05 *vs.* day 0.

To examine whether the inflammatory response persisted after parasite clearance, cytokine levels were measured in intestinal tissues. Increased intestinal IFNγ levels were measured in *Giardia*-infected mice on PI days 7 and 35 compared with uninfected controls (Figure 7A). The intestinal levels of keratinocyte chemoattractant (KC, a homolog of human IL-8) were increased slightly, albeit not significantly, in infected mice on PI days 7 and 35 (Figure 7B). Elevated intestinal TNFα and IL-1β levels were detected in infected mice only on PI day 35 but not day 7 (Figures 7D and 7C). Intestinal IL-6 and IL-12 levels in infected mice did not change at either time point compared with uninfected controls (data not shown).

## Discussion

Accumulating evidence indicates that *Giardia* infection may lead to chronic, post-infective gastrointestinal dysfunction for unknown reasons. The present study detected persistent gut barrier damage and commensal bacterial influx, accompanied by long-term mucosal inflammation, during the post-clearance phase of *Giardia* infection. These novel findings suggest that the host’s unresolved immune response toward its own microbiota, due to an impaired epithelial barrier, may partly contribute to the development of post-infective gut disorders.

In our mouse model of *G. lamblia* infection, peak colonization was detected during the first week, with parasite clearance occurring 2–3 weeks after inoculation. Consistent with other reports, the infection course of *G. lamblia* is much shorter than that of *G. muris* in mice, which lasts for more than 6 weeks [16,21,22]. Instead of murine-specific *G. muris*, a human isolate of *G. lamblia* was used in our model as a zoonotic pathogen to mimic the intestinal pathology in humans. Murine models of *G. lamblia* infection have been utilized to investigate the pathogenesis of villus blunting and epithelial brush border shortening, which are mechanisms responsible for diarrhea, malabsorption, and malnutrition in human patients [23-26].

Based on our observed time course of *G. lamblia* colonization in mice, subsequent experiments were conducted on PI day 7 (colonization period) and PI day 35 (post-clearance phase) to assess gut barrier changes. Consistent with previous studies that demonstrated tight junctional disruptions in giardiasis [9,10,12], we detected occludin cleavage in the jejunum of *G. lamblia*-infected mice during peak parasite colonization. Tight junctional damage in the small intestine persisted during the post-clearance phase, when parasites were no longer detected. Interestingly, colonic tissues, which did not show increased macromolecular flux during peak parasite colonization, displayed heightened epithelial permeabilities after the elimination of parasites. This finding suggests that post-infective host factors may be involved in the mechanism of barrier dysfunction.

During *Giardia* colonization, increased gut-associated bacterial counts were accompanied by enhanced bacterial adherence to and invasion of the small and large intestines, suggesting that parasite infection of the proximal small intestine may lead to bacterial overgrowth and internalization throughout the intestinal tract. Our data indicate that colonization with non-invasive *Giardia* triggered an influx of commensal bacteria, possibly via impaired tight junctions. Recent cell culture studies have suggested that commensal bacteria can pass through paracellular spaces or be engulfed into enterocytes in the presence of other pathogenic invasive bacteria (*e.g. Campylobacteri jejuni* or *Salmonella enterica*) [27,28]. Taken together, colonization by enteropathogens, whether invasive or non-invasive, may affect the luminal confinement of commensals during infection.

The present findings also demonstrate that the phenomena of tight junctional damage and mucosal bacterial invasion persist into the post-clearance phase (at least 5 weeks after the eradication of parasites). It is noteworthy that gut-associated bacterial counts, which mainly include bacteria in the mucus layer, were not higher among post-infective mice, suggesting that increased bacterial penetration may not necessarily correlate with bacterial overgrowth. We identified several bacterial strains (*e.g. Bacillus*, *Staphylococcus*, *Lactobacillus*, and *Phenylobacterium*) in the mucosal endocytosed colonies. Whether these invasive bacteria reflect a compositional change in commensals requires further investigation.

A key role of microbiota in the onset or perpetuation of intestinal inflammation has been implicated in models of enterocolitis using gnotobiotic animals monoassociated with particular bacterial strains or with germ-free versus conventionally raised animals [29,30]. These studies have indicated that chronic gut inflammation may be driven by inappropriate and excessive mucosal immune responses against the host’s own commensal microflora. Aberrant microbiota compositions have been documented in patients with inflammatory bowel disease (IBD) and IBS [31-33]. The emergence of mucosal adherent and invasive bacteria has also been reported in cases of IBD, surgical stress, and necrotizing enterocolitis [34-36]. Whether the composition of commensal bacteria is altered in *Giardia*-infected mouse models remains unknown. Short- and long-term alterations in microfloral diversity associated with giardiasis warrant further investigation.

The activation of immune factors, such as T cells, neutrophils, mast cells, secretory IgA, proinflammatory cytokines, and nitric oxide, have been reported in *Giardia*-infected humans and mice [14,15,22,23,37-39]. A recent report described long-term CD4^+^ T cell proliferative responses in patients 5 years after acute giardiasis [40], suggesting unresolved immune activation during the post-clearance phase. We examined the mucosal inflammatory response in post-infective mouse intestines and identified increased phagocytic infiltration and activation as well as IFNγ and IL-8 production during infection, which persisted into the post-clearance phase. TNFα and IL-1β levels were only upregulated after parasite elimination, whereas no change was detected in IL-6 and IL-12 levels. Previous studies have suggested that the attenuated inflammatory response during *Giardia* infection, which was linked to the activity of parasitic proteases, was a mechanism for *Giardia* to evade host immune responses [41]. We speculate that the post-infective proinflammatory cytokine increase may be caused by secondary bacterial influx in the absence of parasites. The differential patterns of proinflammatory cytokines throughout the course of infection and their relationships with commensal bacterial penetration require further investigation.

Unresolved inflammation also may trigger bacterial influx through either transcellular or paracellular routes. Previous reports have documented tight junctional disassembly and increased paracellular permeability by neutrophil migration [42], or by synergistic effects of IFNγ and TNFα [43]. Recent studies have shown that stimulation with low-dose IFNγ or TNFα was able to induce epithelial internalization and endocytosis of nonpathogenic bacteria [44,45]. Taken together, abnormal bacterial entry and mucosal inflammation may be two events that aggravate each other in chronic post-infective disorders.

## Conclusions

Our results reveal persistent epithelial barrier dysfunction, manifested by tight junctional damage and bacterial influx, following the elimination of *Giardia* parasites. Penetration of multiple strains of commensal bacteria was associated with long-term mucosal inflammation in the absence of parasites. Despite confined colonization of *Giardia* in the proximal small intestine, our findings suggest that epithelial barrier dysfunction and commensal bacterial internalization may be observed throughout the intestinal tract and may persist into the post-clearance phase. Unresolved immune reactions towards the host’s own microbiota may be a major factor contributing to the initiation or perpetuation of post-infective gut disorders. The present study suggests that host-derived factors responding to parasitic infection may induce chronic intestinal barrier dysfunction and commensal bacterial influx. Preventing bacterial penetration by antibiotic treatment or improving epithelial barrier integrity by pharmacological approaches may be beneficial in the management of post-infective disorders following *Giardia* eradication in humans. An understanding of post-infective mechanisms may shed light on the development of novel therapeutic interventions for chronic gut disorders.

## Materials and Methods

### Animals

Specific pathogen free BALB/c mice (4–6 weeks old) were obtained from the Animal Center of National Taiwan University. Animals were raised in a temperature-controlled room (20 ± 2°C) with 12/12-h light/dark cycles and were fed regular mice chow and water *ad libitum*. All experimental procedures were approved by the Animal Care and Use Committee of National Taiwan University.

### *Giardia* infection protocol

Axenic *G. lamblia* GS/M trophozoites (ATCC 50581) were cultured *in vitro* and harvested at log-phase as previously described [14,22]. Mice received 10^7^*G. lamblia* trophozoites suspended in 0.2 ml of sterile phosphate-buffered saline (PBS), or the same volume of PBS alone, by orogastric gavage. To verify the infection time course, mice were sacrificed at various time points (PI day 0, 4, 7, 14, 21, 28, 35, and 49) for the enumeration of trophozoites in the small intestine [8,10]. Briefly, the entire length of the small intestines were opened longitudinally and placed in 10 ml of ice-cold saline for 3 min. Trophozoites were detached from the tissues by vigorous shaking and were counted using a hemocytometer. The detection limit was ~2 × 10^3^ trophozoites per small intestine. Samples of intestinal tissues, liver and spleen were collected for additional analyses on PI days 7 and 35.

### Histological examination

Intestinal segments were fixed in 4% paraformaldehyde and were carefully embedded in paraffin wax to ensure proper orientation of crypt to villus axis. Sections of 4 μm thickness were stained with hematoxylin and eosin (H&E) and observed under a light microscope (Zeiss, Göttingen, Germany). Photoimages were captured using AxioVision Rel. 4.6 software (Zeiss) [46,47].

### Western blotting for occludin cleavage

Scraped intestinal mucosal samples were homogenized in ice-cold complete radio-immunoprecipitation assay (RIPA) buffer, and lysates were sonicated and centrifuged. Supernatant protein concentrations were determined using a protein assay (Bio-Rad, Hercules, CA), adjusted to 5 mg/ml, and diluted 1:1 (v/v) in 2× electrophoresis sample buffer containing 2% (w/v) sodium dodecyl sulfate (SDS), 100 mM dithiothreitol (DTT), and 62.5 mM Tris/HCl (pH 6.8). Samples then were heated to 95°C in a heat block for 5 min and were stored at −20°C until immunoblotting. The extracted proteins were resolved by SDS-polyacrylamide gel electrophoresis, and electrotransferred onto membranes. After blocking with 5% non-fat milk in Tris buffer saline (TBS), membranes were incubated with monoclonal mouse anti-occludin (1:1000; Invitrogen, Carlsbad, CA) at 4°C overnight. Monoclonal mouse anti-β-actin (1:10,000; Sigma, St. Louis, MO) was used to control for equal loading of each sample. Membranes were washed with 0.1% Tween 20 in TBS and were incubated with horseradish peroxidase (HRP)-conjugated goat anti-mouse IgG (1:1000; Cell Signaling, Danvers, MA). Antigens were revealed and band densities were quantified by photoimage analysis [48,49].

### Intestinal permeability assay

Intestinal tissues were opened along the mesenteric border and mounted in Ussing chambers (World Precision Instruments, Sarasota, FL) [46-48]. The opening of each chamber (0.7 cm^2^) exposed the tissue to 5 ml of circulating oxygenated Krebs buffer. The serosal buffer contained 10 mmol/l of glucose that was osmotically balanced with 10 mmol/l of mannitol in the mucosal buffer. A circulating water bath was used to maintain the temperature of the buffer at 37°C. Intestinal permeability was determined as the level of mucosal-to-serosal flux of dextran conjugated to fluorescein isothiocyanate (dextran-FITC, MW = 4 kDa; Sigma). The dextran probe was added to the mucosal buffer at a final concentration of 500 μM. Samples (250 μl) of serosal buffer were collected at 0, 30, 60, and 90 min after addition of the dextran probe and were replaced with Krebs buffer/glucose. The fluorescence units of dextran-FITC in serosal buffer were determined at excitation/emission = 490/530 nm using a multimode plate reader (Paradigm Detection Platform; Beckman Coulter, Indianapolis IN), and dextran-FITC concentrations (nM) were calculated according to a standard curve.

### Measurement of intestinal bacteria numbers

Gut-associated, superficial, and mucosal endocytosed bacterial counts were determined in mice. To evaluate gut-associated bacterial numbers, 1-cm segments of the mid-jejunum and colon were excised and cut longitudinally, and luminal contents were rinsed off with sterile saline. Tissues were weighed aseptically and transferred to PBS at a ratio of 1 mg to10 μl for homogenization and sonication. Tissue lysates were plated onto fresh blood agars (Scientific Biotech, Taipei, Taiwan) for aerobic and anaerobic bacterial culturing overnight at 37°C. Colony-forming units (CFU) were normalized to grams of intestinal tissues (CFU/g) to represent gut-associated bacterial counts [48].

To examine superficial adherent bacterial numbers, 5-cm intestinal segments were excised longitudinally, and luminal contents were rinsed off with sterile saline prior to weighing aseptically. Intestinal tissues were cut into 1-cm pieces and were incubated in 30 ml of sterile PBS with 0.016% DTT (Sigma) at room temperature for 15 min to remove mucus. Tissues were transferred to sterile saline (1:100 w/v) and vortexed for 30 s. Tissues were washed three times with PBS, and the last solution was plated onto fresh blood agars (Scientific Biotech) for aerobic bacterial culturing. CFUs were normalized to grams of intestinal tissues (CFU/g) to represent superficial bacterial counts [50].

To examine mucosal endocytosed bacterial counts, 10-cm-long segments of mid-jejunum (distal of ligament of Triez) and 5-cm segments of colon were cut longitudinally and rinsed in sterile PBS. Tissues were cut into 1-cm portions, incubated in 10 ml of sterile PBS with 1 mM ethylenediaminetetraacetic acid (EDTA) and 5.55 mM glucose (Sigma) at 37°C for 10 min, and then were shaken vigorously. Tissues in solution were passed through nylon mesh (BD Biosciences, San Jose, CA) with a pore size of 80 μm, and single-celled eluates were collected by centrifugation for hemocytometer counting. Our flow cytometry pilot study indicated that 64.0 ± 2.8% of isolated cells were BerEP4^+^ (epithelial marker), 22.0 ± 0.6% were CD68^+^ (macrophage marker), and 38.7 ± 2.0% were CD3^+^ (T cell marker). The amount of intracellular bacteria was determined using a gentamicin protection assay, as described previously [51]. Briefly, single cells were incubated with 300 μg/ml of gentamicin (Invitrogen) for 1 h with gentle shaking to kill extracellular bacteria. Cells then were washed twice with sterile PBS and were incubated with 1% Triton X-100 in PBS for 10 min on ice at a concentration of 5×10^6^ cells/ml. Lysates (200 μl) were plated onto fresh blood agar (Scientific Biotech) and were incubated overnight at 37°C. Bacterial CFUs were normalized to CFU/10^6^ cells to represent mucosal endocytosed bacterial counts.

### Analysis of bacterial translocation to extra-intestinal organs

Livers and spleens were removed from animals using sterile instruments and weighed. Tissues were homogenized and sonicated in sterile PBS at a ratio of 1 mg to 10 μl. Undiluted lysates (200 μl) were inoculated onto Luria-Bertani (LB) agar plates with or without 50 μg/ml ampicillin for aerobic bacterial culturing. Following incubation at 37°C for 24 h, bacterial colonies were counted and normalized to CFU/g [46,48].

### Gram staining

Mucosal endocytosed bacterial colonies grown on agar plates were rapidly classied by Gram staining. Crystal violet was used for peptidoglycan staining, followed by rapid decolorization with alcohol and counterstaining with safranin. All reagents were purchased from Sigma.

### Bacterial 16S rDNA sequencing

Mucosal endocytosed bacterial colonies were picked for DNA extraction using a QIAamp DNA Stool Mini kit (Qiagen, Hilden, Germany). Extracted DNA samples were amplified by polymerase chain reaction (PCR) using a T3000 Thermocycler (Biometra, Göttingen, Germany) with universal bacterial primers detecting the 16S rDNA gene (341F and 534R of *Escherichia coli*) [52]. After amplification, PCR products were sent for sequencing (Genomics BioSci & Tech, Taipei, Taiwan), and sequences were classified using the naïve Bayesian classifier provided by the Ribosomal Database Project (RDP; http://rdp.cme.msu.edu/classifier/classifier.jsp) [53]. Sequences from 16S gene libraries were submitted to the DNA Data Bank of Japan (DDBJ) and were cataloged under the DDBJ accession numbers AB777576–AB777579.

### Fluorescence *in situ* hybridization (FISH)

Microbiological analysis of intestinal tissues was conducted by FISH, with some modifications to previously described protocols [54]. Briefly, tissues were fixed in Carnoy’s solution (Ricca Chemical Company, Arlington, TX), embedded in paraffin, and sectioned at 5 μm. Sections were dewaxed, placed in 1% Triton X-100 for 90 s, washed three times in PBS, and incubated in 5 mg/ml lysozyme at 37°C for 20 min. Sections were washed in PBS three times and were incubated in prewarmed hybridization solution (12% formamide, 20 mM Tris–HCl, 0.9 M NaCl, 0.01% SDS; pH 7.4) containing 0.02–0.1 μM oligonucleotide probes targeting bacterial 16S rDNA at 46°C overnight. The probes included a FITC-labeled universal bacterial probe (EUB-338; 5'-GCTGCCTCCGTAGGAGT-3') and a negative-control probe (Non-338; 5'-CGACGGAGGGCATCCTCA-3') (Genomics BioSci & Tech). Sections then were rinsed in washing buffer (20 mM Tris–HCl, 0.9 M NaCl, 0.01% SDS; pH 7.4) and air-dried prior to staining with Hoechst dye. Slides were mounted and viewed under a fluorescent microscope (Axio Imager A1; Zeiss) equipped with a CCD camera.

### Neutrophil-specific esterase staining of intestinal mucosa

Neutrophils in the intestinal mucosa were detected in paraffin-embedded tissue sections using a naphthol AS-D chloroacetate (neutrophil-specific esterase) staining kit (Sigma) [47]. The substrate, naphthol AS-D chloroacetate, is hydrolyzed by a specific esterase in neutrophils, liberating a free naphthol that couples with a diazonium salt and forms dark purple deposits at sites of enzymatic activity. Tissue sections were stained according to the manufacturer’s instructions, and photoimages were taken using AxioVision Rel. 4.6 software.

### Myeloperoxidase (MPO) activity assay

Intestinal segments were opened longitudinally, rinsed, and weighed. The tissues then were suspended in 0.5% hexadecyltrimethylammonium bromide (Sigma) in 50 mM potassium phosphate buffer (PPB; pH 6) at a ratio of 1 g tissue to 10 ml buffer. Samples then were homogenized and sonicated on ice. Lysates were centrifuged at 12,000 × *g* for 20 min at 10°C, and the resultant supernatants were collected. Supernatants (7 μl) were diluted with 200 μl of reactive buffer (PPB solution containing 0.167 mg/ml of *o*-dianisidine dihydrochloride and 0.0005% of H_2_O_2_) in 96-well plates. Enzyme concentrations were determined as the absorbances at 460 nm measured every 50 s during a 5-min period. One unit of MPO activity was defined as the quantity of enzyme degrading 1 μmol of H_2_O_2_/min at 25°C, expressed as unit/mg tissue [47].

### Total and differential leukocyte counts

Blood was drawn by cardiac puncture with a heparinized syringe into EDTA-containing, pyrogen-free vacutainer tubes (BD Biosciences). Total leukocyte numbers and hematocrit levels in whole blood were determined using a hematology analyzer (Medonic CA-620, Sweden). For differential leukocyte counts, 10-μl blood samples were spread in thin layers on micro-slide glass and stained with Liu’s stain (ASK, Taipei, Taiwan). Differential counts of neutrophils, monocytes, and lymphocytes were determined based on cell morphology under a light microscope.

### Enzyme-linked immunosorbent assay (ELISA)

Jejunal segments (1 cm in length) were weighed and homogenized at a ratio of 1 g tissue to 10 ml PBS which was dissolved with one Complete Mini protease inhibitor cocktail tablet (Roche, Mannheim, Germany). Samples then were sonicated on ice. Lysates were centrifuged at 12,000 × *g* at 4°C for 30 min, and supernatants were collected. Supernatant protein concentrations were measured using a protein assay (Bio-Rad). Cytokine levels in mucosal samples were measured with ELISA development kits according to the manufacturer’s instructions (PeproTech, Rocky Hill, NJ). Briefly, microplates were coated overnight with capture antibodies. Plates were blocked with PBS containing 1% BSA for 1 h and then washed. Sample and standard solutions were added, and plates were incubated for 2 h. Biotinylated antigen-affinity detection antibodies were added, and plates were incubated for an additional 2 h. After washing, an avidin-HRP conjugate was added for 30 min followed by incubation with 2,2'-azino-bis(3-ethylbenzothiazoline-6-sulfonic acid) (ABTS) liquid substrate for color development. Absorbance was measured at 405 nm with correction set at 650 nm. Cytokine levels in intestinal mucosa were expressed as pg cytokine/mg total protein.

### Statistical analysis

All values, except for bacterial CFUs, were expressed as means ± SEM, and means were compared by one-way analysis of variance followed by a Student Newman-Keuls test. For bacterial count data, pairwise rankings of median CFUs were performed using the nonparametric Mann–Whitney *U* test. Differences were considered statistically significant at *P* < 0.05.

## Abbreviations

PI: Post-infective; IBS: Irritable bowel syndrome; PBS: Phosphate-buffered saline; FITC: Fluorescein isothiocyanate; FISH: Fluorescence *in situ* hybridization; CFU: Colony-forming unit; EDTA: Ethylenediaminetetraacetic acid; RIPA: Radio-immunoprecipitation assay; SDS: Sodium dodecyl sulfate; DTT: Dithiothreitol; IBD: Inflammatory bowel disease; RDP: Ribosomal Database Project; DDBJ: DNA Data Bank of Japan.

## Competing interest

The authors declare that there are no conflicts of interest.

## Authors' contributions

Guarantor of integrity of entire study, LCY; study concepts and design: LCY and AGB; data acquisition: TLC, HC, HWW, YZL, and LLW; data analysis/interpretation: TLC, HC, HWW, and TCL; statistical analysis: TLC, HWW; material and technical support: YHN, CHS, WHY; obtained funding: LCY; manuscript drafting or revision for important intellectual content, literature research, manuscript editing, and manuscript final version approval: all authors.

## Supplementary Material

Additional file 1: Figure S1 Normal mucosal morphology in mouse jejunum and colon samples during colonization and post-clearance of *Giardia* infection. Histological images of mouse jejunal and colonic tissues by H&E staining (magnification 200×). Mucosal morphologies in infected mice were not altered compared with saline controls on PI days 7 or 35. *n* = 6/group. Click here for file

Additional file 2: Figure S2 Persistent anaerobic bacterial overgrowth in the intestines of *Giardia*-infected mice. Gut-associated bacterial counts were determined in jejunum **(A)** and colon **(B)** tissues on PI days 7 and 35 using anaerobic culture conditions. Each data point represents one animal. Bars indicate the median bacterial counts. *n* = 6–7/group. *P < 0.05 *vs.* PBS. Click here for file
