# Supplementary figures and images for: Persistent gut barrier damage and commensal bacterial influx following eradication of Giardia infection in mice
Source: Gut Pathog. 2013 Aug 30;5:26. doi: 10.1186/1757-4749-5-26 (PMC3765889; doi:10.1186/1757-4749-5-26)

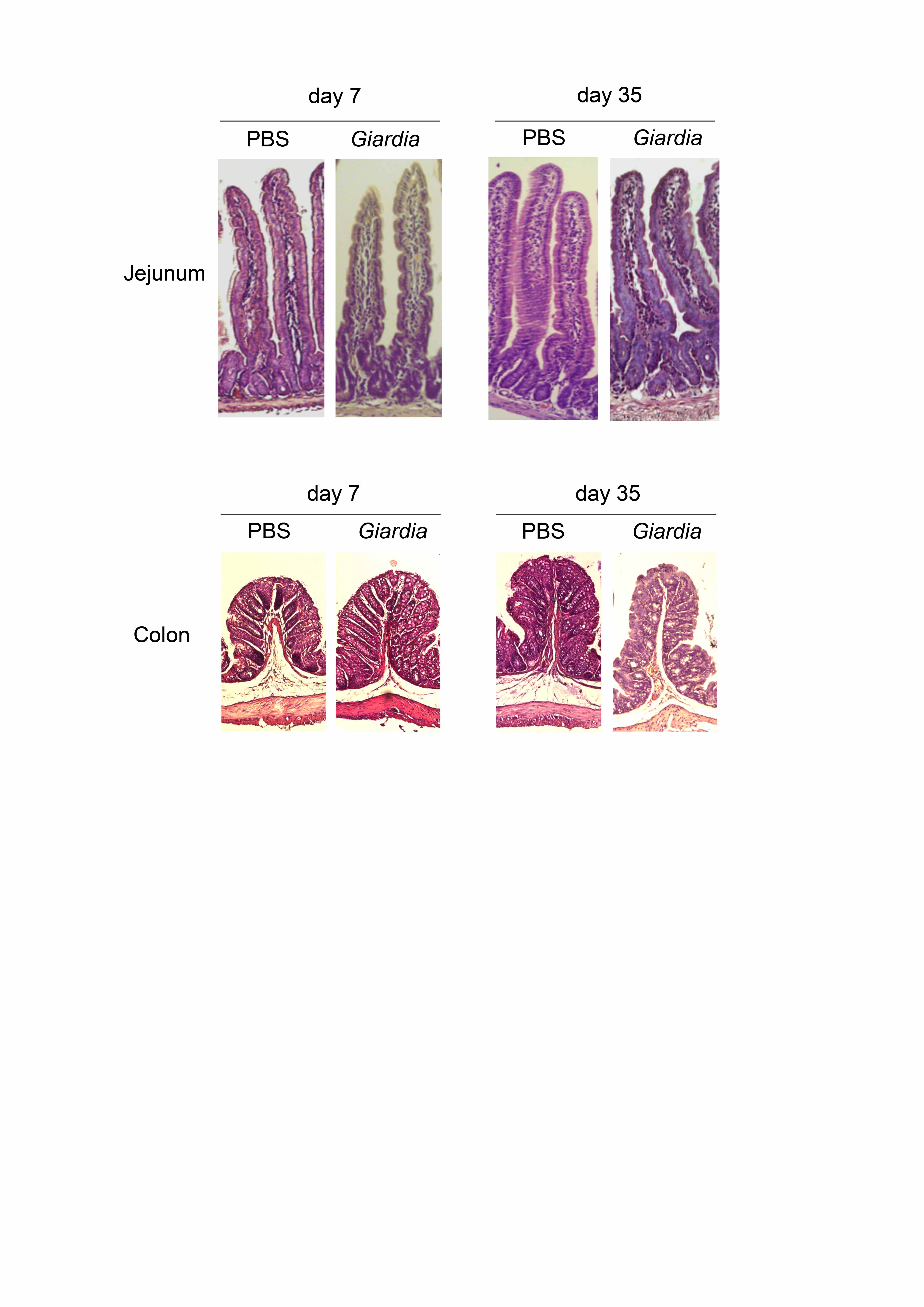

Supplement: Additional file 1: Figure S1 — Normal mucosal morphology in mouse jejunum and colon samples during colonization and post-clearance of Giardia infection. Histological images of mouse jejunal and colonic tissues by H&E staining (magnification 200×). Mucosal morphologies in infected mice were not altered compared with saline controls on PI days 7 or 35. n = 6/group. [file 1757-4749-5-26-S1.jpeg]

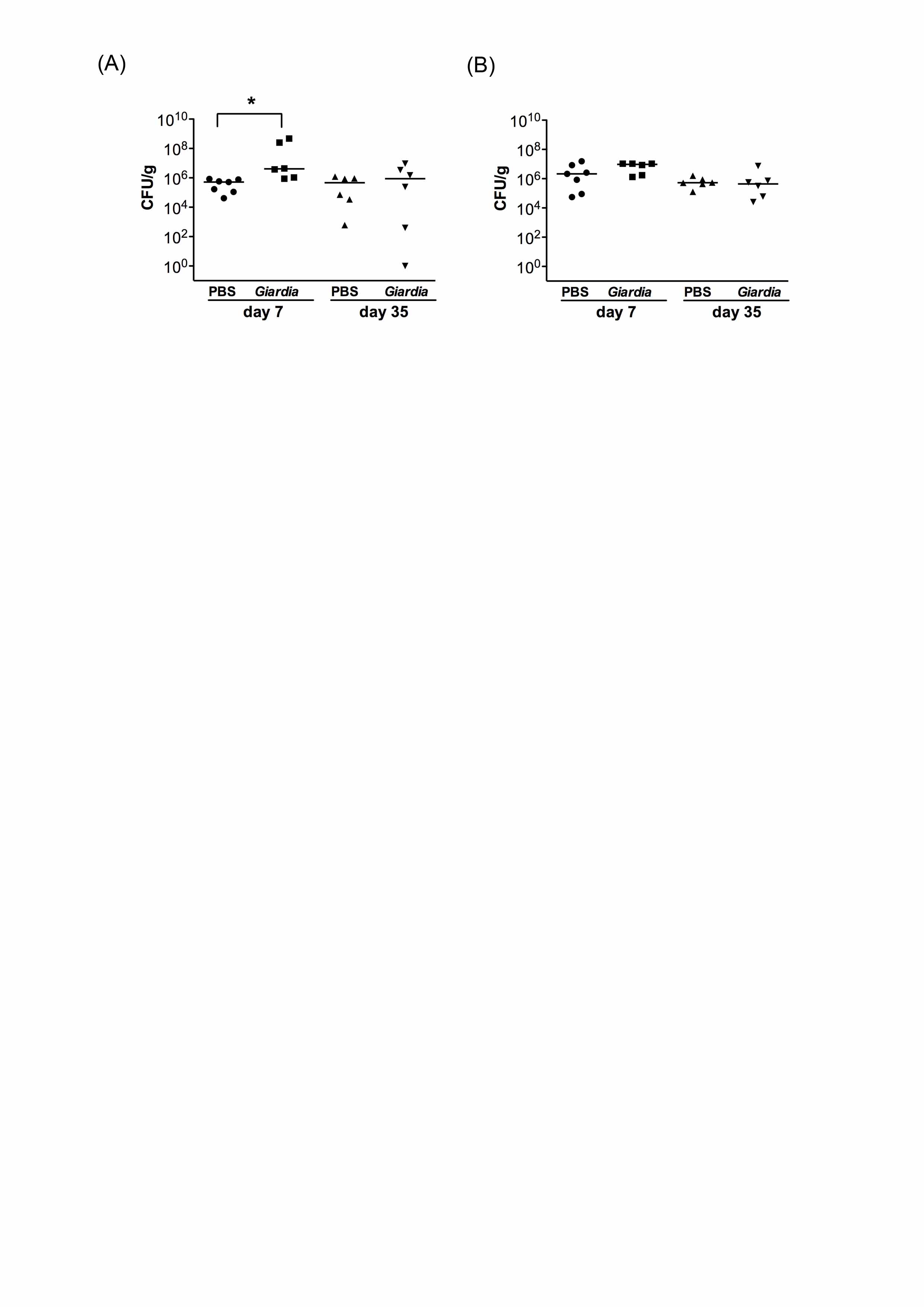

Supplement: Additional file 2: Figure S2 — Persistent anaerobic bacterial overgrowth in the intestines of Giardia-infected mice. Gut-associated bacterial counts were determined in jejunum (A) and colon (B) tissues on PI days 7 and 35 using anaerobic culture conditions. Each data point represents one animal. Bars indicate the median bacterial counts. n = 6–7/group. *P < 0.05 vs. PBS. [file 1757-4749-5-26-S2.jpeg]
